# Clinical signs of bruxism in CrossFit^®^ practitioners: observational study

**DOI:** 10.1590/2177-6709.29.5.e242476.oar

**Published:** 2024-10-04

**Authors:** Igor Ferreira Batista RIBEIRO, Karina Miranda LIEUTHIER, Gabriela De Sena FERREIRA, Vanara Florêncio PASSOS, Raniel Fernandes PEIXOTO, Sandra Maria Abreu NOGUEIRA, Paula Jordani ONGARO, Lívia Maria Sales Pinto FIAMENGUI

**Affiliations:** 1 Federal University of Ceará, School of Dentistry (Fortaleza/CE, Brazil).; 2 Federal University of Ceará, School of Dentistry, Department of Restorative Dentistry (Fortaleza/CE, Brazil).; 3 Private practice (Araraquara/SP, Brazil).

**Keywords:** Bruxism, High-intensity interval training, Clinical signs, Bruxismo, Treinamento intervalado de alta intensidade, Sinais clínicos

## Abstract

**Introduction:**

CrossFit® is a physical training method that aims to promote physical fitness through the development of components such as aerobic capacity, strength and muscular endurance. Data regarding bruxism behaviors in CrossFit® practitioners are scarce, but previous studies have shown increased dental clenching behavior during weightlifting practices.

**Objective:**

The present study aimed to evaluate clinical signs of bruxism in CrossFit® practitioners.

**Methods:**

The sample comprised a convenience sample of CrossFit® practitioners (n=57), of both genders, aged 19-58 years. Outcome variables were as follows: Oral Behavior Checklist, the International Physical Activity Questionnaire and the Standardized Tool for the Assessment of Bruxism. Data were expressed in terms of absolute values and percentages. Spearman’s correlation and Fisher exact tests were used for statistical analysis, and a significance level of 5% was considered.

**Results:**

The mean age was 32.82 years, with a female majority (63.15%). CrossFit® practitioners frequently reported clenching their teeth during training practice (61.40%) and presented linea alba (82.45%), lip impression (54.38%), tongue impression (26.31%), bone exostosis (19.29%), tooth wear (61.40%) and non-carious cervical lesions (35.09%).

**Conclusions:**

No correlation was found between clinical signs of bruxism, oral behaviors and physical activity intensity; however, the results suggest that individuals who practice CrossFit® training have oral behaviors that can cause morpho-functional changes in the stomatognathic system, especially the habit of clenching their teeth during training. In addition, data emphasize the need for dental health education among CrossFit® practitioners, and more studies with a representative sample are necessary.

## RESUMO

**Introdução:**

O CrossFit^®^ é um método de treinamento físico que visa promover a aptidão física por meio do desenvolvimento de componentes como capacidade aeróbia, força e resistência muscular. Os dados sobre comportamentos de bruxismo em praticantes de CrossFit^®^ são escassos, mas estudos prévios mostraram aumento do comportamento de apertamento dentário durante as práticas de levantamento de peso.

**Objetivo:**

O presente estudo teve como objetivo avaliar os sinais clínicos de bruxismo em praticantes de CrossFit^®^.

**Métodos:**

O estudo foi composto por uma amostra de conveniência de praticantes de CrossFit^®^ (n=57), de ambos os sexos, com idade entre 19 e 58 anos, e as variáveis de desfecho foram: Lista de Verificação de Comportamento Oral, Questionário Internacional de Atividade Física e Ferramenta Padronizada para Avaliação do Bruxismo. Os dados foram expressos em termos de valores absolutos e percentuais. Para análise estatística, foram utilizados os testes de correlação de Spearman e exato de Fisher, e considerou-se nível de significância de 5%.

**Resultados:**

A média de idade foi de 32,82 anos, com maioria feminina (63,15%). Os praticantes de CrossFit^®^ relataram frequentemente apertar os dentes durante a prática do treinamento (61,40%) e apresentaram linha alba (82,45%), impressão labial (54,38%), impressão de língua (26,31%), exostose óssea (19,29%), desgaste dentário (61,40%) e lesões cervicais não cariosas (35,09%).

**Conclusões:**

Não foi encontrada correlação entre sinais clínicos de bruxismo, comportamentos orais e intensidade da atividade física; no entanto, os resultados sugerem que indivíduos que praticam o treinamento CrossFit^®^ apresentam comportamentos orais que podem causar alterações morfofuncionais no sistema estomatognático, principalmente o hábito de apertar os dentes durante o treinamento. Além disso, os dados enfatizam a necessidade de educação em saúde bucal entre os praticantes de CrossFit^®^, sendo necessários mais estudos com amostras representativas.

## INTRODUCTION

CrossFit^®^ is a physical training method that seeks to promote physical fitness through the development of components such as aerobic capacity, muscular strength and endurance, speed, coordination, agility and balance,^1^ by means of functional exercises, weight lifting, gymnastic movements, and aerobic conditioning, which can be performed at high intensity.^2^

Resistance training, or strength training (ST), such as weight lifting, has been recognized as an important component of the physical conditioning program for adults, due to the promotion of several health benefits, mainly as prevention of several diseases.^3^ Its benefits range from reducing the risk of premature death, heart disease, stroke and type II diabetes to reducing stress, anxiety and depression, as well as promoting well-being.^4^

Previous studies have investigated the correlation between stomatognathic system and sports performance. It has been suggested that jaw clenching, with or without a mouthguard, promotes enhanced neuromuscular performance parameters.^5^ In addition, practitioners often report deleterious habits, such as clenching their teeth while exerting muscular effort during tasks, such as lifting some heavy weight or participating in a sport that requires maximum effort.^6^ ^,^ ^7^ Although there is some improvement in performance, few studies have evaluated the impact of clenching on soft tissue and dental structure during strength training. The American College of Sports Medicine (ACSM) highlights the injury risks involved in performing some exercises that, if done incorrectly or excessively, can cause some musculoskeletal and/or orofacial injuries.^8^

It has been recognized that bruxism cannot be considered a disorder or a primary disease, but rather a repetitive muscular activity characterized by clenching or grinding the teeth and/or supporting or pushing the jaw. Some of these behaviors may be found in the resistance training of CrossFit^®^ practice, being considered risk factors for alterations in the stomatognathic system, and may be the cause of, for example, mechanical wear of the teeth and pain in the muscles of mastication and temporomandibular joint. Bruxism can be manifested in two distinct circadian forms: it can occur during wakefulness (awake bruxism, AB) or during sleep (sleep bruxism, SB).^9^

Literature is still quite scarce on the pathophysiology of bruxism during wakefulness, although it is known that there is a proportional association with increased muscle activity and emotional stress (daily psychosocial tension).^10^ The literature shows that this behavior can occur in approximately 16-32% of the population, which is not related to gender,^11^ but it presents a multifactorial etiology, with interaction between biological, psychosocial, genetic, environmental and lifestyle factors.^12^ ^,^ ^13^

Clinically, signs of bruxism may involve the presence of hypertrophy of the masticatory muscles, indentations on the tongue or lip, and evident linea alba on the jugal mucosa.^14^ Damage to hard dental tissues such as tooth wear (TW), repetitive failures of restorative treatments, formation of dental abfractions, and an increased likelihood of pain in the muscles of mastication and temporomandibular joint are also reported in the literature.^9^ In order to assess bruxism in a multifactorial and standardized way, the Standardized Tool for the Assessment of Bruxism (STAB) suggests self-report analyses, together with clinical and instrumental assessment.^15^

Some studies relate jaw clenching muscle activities during wakefulness to the moment of concentration and strength, which may reflect the perception of effort during incremental workload cycling.^5^ In contrast, there is no information about the possible clinical consequences of that behavior. Given the scarcity of solid information on the subject, it is of paramount importance that studies are developed in this area seeking, in the future, the consolidation of preventive measures and protocols for the management of bruxism during sports.

Therefore, seeking to identify the relationship between the moment of physical activity and awaking bruxism, the present study aimed to evaluate clinical signs of bruxism in CrossFit^®^ practitioners. 

## MATERIAL AND METHODS

This observational study was approved by the Ethics Committee of the Federal University of Ceará (PROPESQ/UFC, CAAE number: 71612723.0.0000.5054), informed written consent was obtained from the participants, and all methods were carried out in accordance with Helsinki guidelines and regulations.

Eligible participants included those aged above 18 years, of both genders, practicing CrossFit^®^ training at least three times a week, for at least six months. Exclusion criteria included previously diagnosed disabling cognitive, psychological and neurological disorders, history of previous bruxism management, individuals being treated with antidepressants, drug users, and/or alcohol abuse, as well as pregnant and lactating women. Also, those who did not agree to participate were not included.

A convenience sample was used due to the impossibility of calculating a representative sample, as no list rolling all practitioners was available.

The study was conducted by a single examiner, who performed clinical examination and questionnaire applications. Data regarding sociodemographic characteristics, such as gender, age, ethnicity, employment, marital status and education level, were also collected.

Oral behaviors (OB) are often detected by patients’ self-reports, using instruments such as questionnaires or checklists. Oral Behavior Checklist (OBC) is a valid instrument to quantify the frequency of OB during sleep and wakefullness.^16^ It aims to assess a series of harmful habits reported during the last 30 days. In the full version, it consists of 21 questions, 2 of which are about habits during sleep (how many nights in a week such behavior appears) and 19 about habits during wakefulness (none of the time/a little of the time/some of the time/most of the time/all of the time). For each item, a score of 0-4 points is assigned, yielding a total sum in the range from 0 to 84 points. The score is interpreted as follows: 0, no risk of parafunctional oral activity; 1-24, low risk of parafunctional oral activity; 25-84, high risk of parafunctional oral activity.^16^ In addition, an extra question was added, in which the participants were asked to answer whether they clenched their teeth during training.

The International Physical Activity Questionnaire (IPAQ) is a standardized self-report questionnaire and it is commonly used as a measurement tool. IPAQ can provide researchers and practitioners with an estimate of physical activity and sedentary behavior for adults aged 15-69 years, and it has already been shown to be a valid tool for this purpose. This questionnaire classifies the level of intensity of physical activity as highly active, active, irregularly active, and sedentary.^17^

For volunteers who have practiced vigorous activity for five days or more for at least half an hour, they are classified as very active. Those who performed vigorous activity for three days or more for at least twenty minutes per session are classified as active. The other classifications were not included in this study, because they were part of the exclusion criteria.

IPAQ estimates the time spent weekly in physical activity practices of different intensities, during work, transportation, household chores and leisure, and also the time spent in inactive activities, performed in the sitting position. The short version of the physical activity questionnaire that was used presents eight questions related to the activities performed in a normal week, with vigorous or moderate intensity, lasting ten minutes continuously, in different contexts (work, transportation, domestic activities and leisure).^17^

The Standardized Tool for the Assessment of Bruxism (STAB) is a classification system proposed by a group of experts to establish a framework for validating different approaches. This clinical evaluation study was performed based on Axis A (Assessment of Bruxism Status and Consequences) of the STAB criteria, related to the Clinical Basis Assessment (CBA), which includes domains on intraoral and extraoral tissues (evaluation of the presence of several signs, such as linea alba, lip impression, tongue scalloping, tongue traumatic lesion, alveolar bone exostosis) and teeth and restorations, performing the quantification and qualification of dental wear, based on the Tooth Wear Evaluation System (TWES),^18^ with an analysis of the influence of mechanical factors on clinical signs, such as the location and proportion of the lesion. The TWES analyses the six sextants, and each of the occlusal/incisal surfaces are graded using a 5-point ordinal occlusal/incisal grading scale (0 = no wear; 1 = wear confined to enamel; 2 = wear with exposed dentin ≤1/3 of crown height; 3 = wear >1/3 but ˂2/3 of crown height; 4 = wear ≥2/3 of crown height). Additionally, the second sextant was also graded at the palatal surfaces, because these surfaces play a significant role in the articulation as well.

Data obtained from the questionnaire and intraoral examination were used to characterize the sample. The data were subjected to descriptive analysis and hypothesis tests, such as the chi-square test or Fisher’s exact test. When the expected frequencies were less than 5, the Fisher test was used to obtain more precise and reliable results (SPSS 22.0, IBM Corp., Chicago, USA). These tests were used to analyze the association between clinical signs of bruxism with reported oral behaviors and the intensity of physical activity. The significance level was 95% (*p*< 0.05) for all analyses.

## RESULTS

A total of 57 volunteers were included in the study. The mean age was 32.82 years (ranging from 19 to 58 years). The characterization of the sample is described in Table 1. Most of the sample was comprised of female gender (63.15%), aged 30 or over (56.14%), and white ethnicity (64.91%). The mean score was 21.26 points in the OBC standardized test. Thirty-nine participants (66.67%) were classified as showing low-risk level, against 18 (33.33%) in the high-risk level of OB. Regarding the extra question in OBC questionnaire, 35 (61.40%) reported clenching the teeth during training.

**Table 1:** Sample characterization.

Variables	n (%)
Sex	
Female	36 (63.15)
Male	21 (36.84)
Age	
< 30 years	25 (43.85)
≥ 30 years	32 (56.14)
Ethnicity/skin color	
White	37 (64.91)
Mixed	15 (26.31)
Black	4 (7.01)
Indigenous	1 (1.75)
“Clench the teeth during training?”	
Yes	35 (61.40)
No	22 (38.59)
OBC	
Low risk	38 (66.67)
High risk	19 (33.33)

There were no volunteer classified as “no risk” in OBC classification.

Considering physical activity data, most of the sample (71.92%) reported practicing CrossFit^®^ 4-6 times/week, and 37 participants (64.91%) reported a training duration of 30-60 minutes. According to the IPAQ, most of the sample (87.71%) had a level of physical activity classified as highly active (Table 2).

**Table 2:** Characterization of sports practice.

Variables	n (%)
IPAQ	
Highly active	50 (87.81)
Active	7 (12.28)
Frequency (per week)	
3 days	9 (14.03)
4-6 days	41 (71.92)
Everyday	7 (12.28)
Duration (minutes)	
30-60	37 (64.91)
60-90	16 (28.07)
Above 90	4 (0.07)

Furthermore, analyzing the clinical signs of bruxism, the participants presented: linea alba (82.45%), lip impression (54.38%), tongue impression (26.31%), bone exostosis (19.29%), tooth wear (61.40%) and non-carious cervical lesions (NCCL) (35.09%) (Table 3).

**Table 3:** Clinical signs of bruxism, according do STAB Axis A.

Clinical sign	n (%)
Linea alba	
Yes	47 (82.45)
No	10 (17.54)
Lip impression	
Yes	31 (54.38)
No	26 (45.61)
Tongue impression	
Yes	15 (26.31)
No	42 (73.68)
Bone exostosis	
Yes	11 (19.29)
No	46 (80.70)
Tooth wear	
Yes	35 (61.40)
No	22 (38.59)
NCCL	
Yes	20 (35.09)
No	37 (64.91)

*NCCL = non-carious cervical lesion.

As presented on Table 4, clinical signs of bruxism was not correlated to OBC score or IPAQ classification.

**Table 4:** Correlation of bruxism clinical signs to OBC score and IPAQ classification.

Variables	OBC				IPAQ		
	Total (n)	High	Low	p-value	Highly active	Active	p-value
Linea alba							
Yes	47	16	31	1.00*	41	6	1.00*
No	10	3	7		9	1	
Lip impression							
Yes	31	11	20	0.707^#^	26	5	0.436^#^
No	26	8	18		24	2	
Tongue impression							
Yes	15	6	9	0.523^#^	13	2	1.00*
No	42	13	29		37	5	
Bone exostosis							
Yes	11	2	9	0.304*	10	1	1.00*
No	46	17	29		40	6	
Tooth Wear (TW)							
Yes	35	11	24	0.700^#^	31	19	1.00*
No	22	8	14		4	3	
Non-carious cervical lesions							
Yes	20	8	12	0.432^#^	18	2	1.00*
No	37	11	26		32	5	

TW+ (Patients with wear level ≥2). ^#^Chi-squared test. *Fisher’s exact test.

## DISCUSSION

To the best of our knowledge, this observational study was the first one aiming to evaluate the prevalence of clinical signs of bruxism in CrossFit^®^ practitioners by using the Axis A of the STAB. Despite the increasing interest in CrossFit^®^ in the recent years, there is still a gap concerning the related injury risks and bruxism behaviors during its practice. The present findings showed high frequency of linea alba (82.45%), tooth wear (61.40%), and lip impression (54.38%). NCCL, tongue impression and bone exostosis occurred in 35.09%, 26.31% and 19.29% of the participants, respectively. Most of the participants (61.40%) reported clenching their teeth during CrossFit^®^ training. There was no correlation between clinical signs of bruxism (tooth wear, NCCL, linea alba, lip impression, tongue impression and bone exostosis) and the OBC scores and IPAQ classification. 

A previous study evaluated the maximum molar bite force and thickness of the masseter and temporal muscles of athletes. The findings include that the CrossFit^®^ group showed greater molar bite force than the group that did not practice the sport. Regarding muscle thickness, there were significant differences for the right (p=0.032) and left (p=0.004) masseter muscles in dental clenching in maximum voluntary contraction, when compared to control group.^19^ However, there is a lack of studies aiming to evaluate signs of bruxism, and the main focus remain on injury occurrence, showing high prevalence of impacts on the mental protuberance, upper lips and upper teeth.^20^

With this in mind, the STAB was created with the aim of reducing the clinical difficulties in assessing bruxism, with more comprehensive analyses based on the multifactorial nature of this behavior. However, as it is a new standardization proposal, there is still a need for further studies to validate this tool.

In the present study, data from sedentary individuals or from those practicing another physical activity were not collected, thus, interpreting data presented here may be challenging. Previous study found clinical signs of bruxism in 47% of their sample, such as tongue indentation(s), tooth wear and/or bone exostosis among athletes of swimming, athletics, gymnastics and tennis.^21^ Another study observed that high-performance kayak and canoe athletes are predisposed to bruxism and suffer from various pathologies related to it. It is hard to define that these clinical signs are caused by the behaviors during training practice, but 11.17% of the athletes complained having suffered tooth fractures during training. Additionally, they reported an increase in bone exostosis in both kayak (58.8%) and canoe (26.4%) groups, suggesting that excessive forces may lead to an increased volume of the alveolar bone.^22^ Another study evaluated the presence of NCCL among soccer, rugby, volleyball, handball and fighting sports athletes, in which 17.42% presented NCCL. Its prevalence rose with increasing age and training time.^23^ During the London 2012 Olympic Games, dental erosion was highly prevalent (45%) among 278 elite athletes.^24^

One must keep in mind is that the etiology and progression of these clinical signs analyzed in this study are multifactorial and involve complex interactions associated, for example, with the concentration of cervical stress, mechanical friction, endogenous and exogenous acids and, also, sleep and awake bruxism behaviors, making its diagnostic and associations harder to assess.^25^ ^,^ ^26^

The present study has limitations. First, it was an observational study and there were sample calculation issues due to the impossibility of calculating a representative sample, as no list rolling all practitioners was available until this research. Secondly, the STAB is a new standardization proposal, so there is still a need for further studies to validate this tool. For future research, longitudinal studies, presence of a control group and representative samples are needed in order to better elucidate bruxism signs and symptoms among CrossFit^®^ practitioners and its impact to the stomatognathic system. For instance, dental health education among CrossFit^®^ practitioners is suggested to manage signs and symptoms of bruxism possibly related to sports practice.

## CONCLUSION

Despite the limitations of this study, the results suggest that individuals who practice CrossFit^®^ training have oral behaviors that can cause morpho-functional changes in the stomatognathic system, especially the habit of clenching their teeth during training. This behavior was exhibited by 61.40% of the sample, but, in addition, a significant proportion presented linea alba, lip impression, tooth wear and non-carious cervical lesion, which emphasizes the need for dental health education among CrossFit^®^ practitioners.

Although no statistically significant correlations were found, future research is necessary to further explain the present findings, which shows that there are still gaps in sports dentistry when evaluating the dynamic structures of the stomatognathic system associated with high-intensity sports modalities.
